# Effect of deep margin elevation on fracture resistance of premolars restored with ceramic onlay: *In vitro* comparative study

**DOI:** 10.4317/jced.60384

**Published:** 2023-06-01

**Authors:** Zahraa Salah, Ahmed Sleibi

**Affiliations:** 1 MSc researcher, Department of Conservative Dentistry, College of Dentistry, Mustansiriyah University, Baghdad, Iraq. Health Center Al-Taji first, Department health of Baghdad Karkh, Iraqi Ministry of Health; 2 Department of conservative dentistry, College of Dentistry, Mustansiriyah University, Baghdad, Iraq

## Abstract

**Background:**

This *in vitro* comparative study aimed to compare the influence of two levels of deep margin elevation (2 and 3 mm) with either bulk-fill flowable composite or short fiber-reinforced flowable composite on the fracture resistance of maxillary first premolars restored with ceramic onlays.

**Material and Methods:**

Fifty sound-extracted maxillary first premolar teeth were selected to prepare mesio-occluso-distal cavities with standardized dimensions. The cervical margins were extended 2 mm below the cemento-enamel junction on both mesial and distal sides. These teeth were randomly divided into five groups: Group I: no box elevation (control group). Group II: 2 mm marginal elevation with bulk-fill flowable composite. Group III: 2 mm marginal elevation with short fiber-reinforced flowable composite. Group IV: 3 mm marginal elevation with bulk-fill flowable composite. Group V: 3 mm marginal elevation with short fiber-reinforced flowable composite. After cementation, all teeth were subjected to a fracture resistance test using the universal testing machine, and the mode of failure was analyzed using a digital microscope at 20x magnification.

**Results:**

The result showed a non-significant difference in the fracture resistance between 2 and 3 mm marginal elevation (*p*>0.05) with respect to each restorative material used for deep margin elevation. However, the fracture resistance of teeth elevated with short fiber-reinforced flowable composite was significantly higher than those elevated with bulk-fill flowable composite at both levels 2 and 3 mm, *p*=0.041 and 0.038 respectively.

**Conclusions:**

The fracture resistance of premolars restored with a ceramic onlay was not influenced by the levels of deep margin elevation (2 or 3 mm). However, marginal elevation with short fiber-reinforced flowable composites provided higher fracture resistance than those elevated with bulk-fill flowable composites, and those without marginal elevation.

** Key words:**Fracture Resistance, Short fiber reinforced flowable composite, Bulk-fill flowable composite, Ceramic onlay, Cervical margin elevation.

## Introduction

Restoration of large cavities with weak remaining tooth structure is a case of challenge in dental practice (1). Cuspal reduction of 1.5-2 mm was recommended, followed by indirect bonded restoration to increase the fracture resistance (1). The margins of these large cavities often extend subgingivally, making isolation, impression, and subsequent adhesive cementation of clinical concern. Dietschi and Spreafico (1998) suggested that the placement of a composite layer could transform the subgingival margin into a supragingival position. This procedure was named “Deep Margin Elevation (DME)” (2). However, questions remained regarding the recommended thickness and the suiTable restorative material that could be placed beneath the milled restoration that resists the occlusal load.

In terms of restoration longevity, fractures and secondary caries have been shown to be the primary causes of failure in ceramic posterior restorations (4). According to the studies, caries is the result of long-term failure, whereas fractures are more related to early failure (5). A long-term study with a follow-up of more than 10 years also concluded that failure was more closely associated with fracture compared to caries (6).

All-ceramic restoration was considered an excellent treatment option for treating posterior teeth with a considerable loss of tooth structure and an esthetic requirement. This is due to their aesthetic characteristics, color stability, chemical toughness, fluorescent properties, wear resistance, and biocompatibility in the oral environment (7). Additionally, by incorporating fillers like lithium disilicate and alumina into the glass matrix, the physical qualities of all ceramic restorations have been enhanced, leading to stronger and more fracture-resistant restorations (8). Lithium-silicate ceramics was reported to have a positive clinical outcome in follow-ups with a relatively high success rate of 93% (8).

Despite the material properties of Lithium-silicate ceramics, the thickness of ceramic restorations was considered an important factor in fracture resistance (9). Previous research concluded that the stress exerted on the remaining tooth structure increases with increasing the depth of the restoration (10,11). A previously published study also showed that inlays with shorter occluso-cervical height following DME would be the least associated with ceramic fracture and restoration failure (12).

Bulk-fill (BF) composites might be considered the material of choice for DME due to their enhanced consistency, ease of placement, and little instrument pullback (3). In addition, they have a greater depth of cure that reaches 4-5 mm in thickness (13). This would be achieved by means of new resins, a changed initiator mechanism, polymerization stimulators, unique fillers, and filler control, all of which led to an improvement in their clinical status (14,15). However, their mechanical properties remained questionable for use in high-stress areas under indirect restoration.

A new experimental restorative material known as short fiber-reinforced flowable composite (SFRC) was recently introduced to the market (15). This type of composite material was suggested for use as a dentine replacement material in high-stress locations, particularly in extensive cavities of posterior teeth. It is composed of a resin matrix, randomly oriented glass microfibers, and inorganic silanated particle fillers, and is available in bulk shades with a 5 mm depth of cure (15). Previously published studies showed improved flexural toughness of SFRC compared to flowable BF composites and other conventional particulate-filled resin composites in high-stress areas (16).

Nevertheless, limited data is available to assess the impact of using the SFRC to elevate the subgingival margin with regard to the reinforcement of tooth and/or restoration. The aim of this study was to investigate the influence of two levels of DME (2 and 3 mm) with either bulk-fill flowable composite or short fiber-reinforced flowable composite on the fracture resistance of maxillary premolars restored with ceramic onlay using a universal testing machine. The first null hypothesis of this study is the fracture resistance of maxillary premolars restored with ceramic onlays and deep margin elevation is not influenced by the level of elevation (2 mm or 3 mm) or the type of composite material used. The second null hypothesis states that the mode of fracture of maxillary premolars restored with ceramic onlays and deep margin elevation is unaffected by elevation of 2mm or 3mm or by the type of composite material.

## Material and Methods

The materials utilized in the present study are listed in Table 1. Ethical approval (No. MUOPR12) was obtained to collect fifty sound maxillary first premolars extracted for orthodontic purposes, ages 18-25. These teeth had comparable size, dimensions, and occlusal anatomy. The total sample size was 50, and each group had 10 samples. The sample size calculation was applied with respect to the previous studies (12,17). At all stages of the study, dehydration of the teeth was avoided. Each tooth was placed inside a cold-cured acrylic block, at a level of 3 mm apical to the cemento-enamel junction (CEJ). Silicon light body impression material was then injected between the embedded root and acrylic, which replace the 0.2-0.3 mm thickness of the wax layer that had already been placed prior to the cold-cured acrylic to simulate the periodontal ligament (18).

**Table 1:**
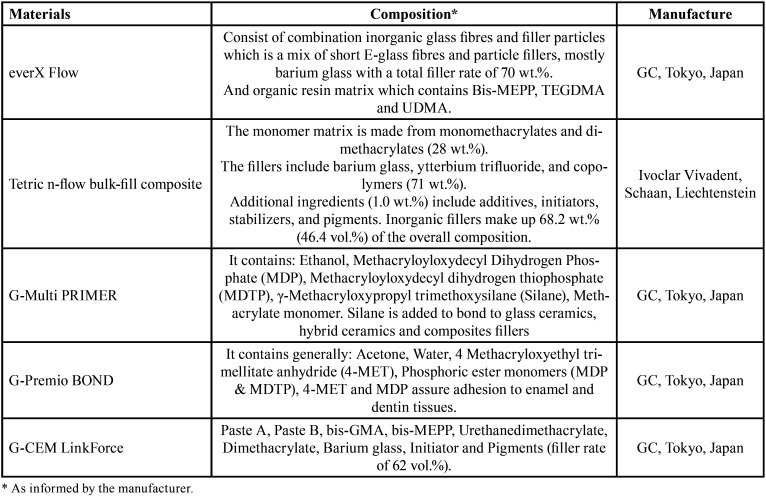
Materials that have been utilized in the present study.

**Figure 1:**
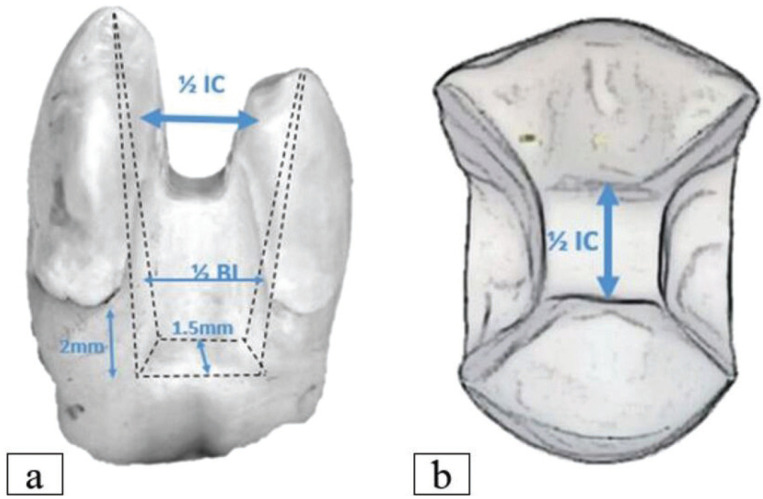
Diagram illustrates the dimensions of cavity preparation: (a) proximal view, (b) occlusal view.

**Figure 2:**
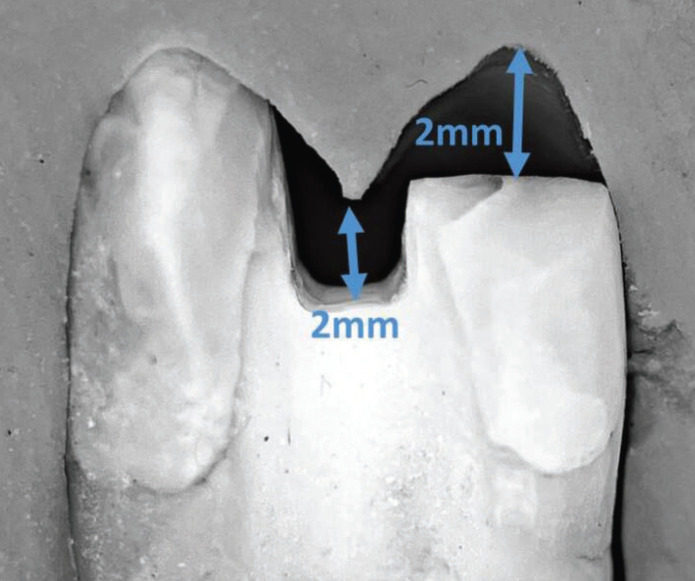
Checking the preparation with the silicon index (the black background).

To control the dimensions of cavity preparation, a silicon index was made from heavy body impression material for each tooth, which was then sectioned buccolingually (19). The dimensions of cavity preparation were drawn on the samples using a digital caliper and marker (20). The preparation was made using an inlay preparation kit (REF 4562, Komet, Lemgo, Germany) with the following dimensions: The occlusal cavity was prepared at a depth of 2 mm from the level of the central groove, and the width was half the intercuspal distance. This was done using a cylindrical diamond bur (No. 959KRD, 314, 018, Komet, Lemgo, Germany). Proximal boxes were prepared using a tapered diamond bur (No. 6847KRD, 314,016, Komet, Lemgo, Germany), with ½ buccolingual dimensions and 1.5 mm at the gingival floor in a mesiodistal direction (21). The cervical margins were placed 2 mm below the CEJ on the mesial and distal sides. A conical diamond bur (No. 8379, 314, 023, Komet, Lemgo, Germany) was used to reduce the palatal cusp by 2 mm in accordance with the anatomical contour of the occlusal surface (19). For every four preparations, a new bur was used to ensure the cutting efficiency (19). A silicone index and a periodontal probe were used to control and measure the depth of preparation. All preparations were done with a 3x magnification loupe (eighteeth, Changzhou, China) (Figs. 1,2).

The samples were then randomly divided into five groups (n=10 each) according to the level of DME and the type of restorative material used. Group I: No box elevation, where the cervical margin was kept 2 mm below the CEJ and considered the control group. Group II: 2 mm of DME with flowable BF composite. Group III: 2 mm of DME with SFRC. Group IV: 3 mm of DME with flowable BF composite. Group V: 3 mm of DME with SFRC.

To avoid the overfilling, a Tofflemire matrix band was used before the restoration placement at the level of 2 or 3 mm, with respect to the gingival seat. The allocated area of elevation in both mesial and distal boxes was treated with 15 s of selective acid etching using 37% phosphoric acid (Ivoclar Vivadent, Schaan, Liechtenstein), rinsed for 10 s, then gently air dried for 5 s using a 3-in-1 dental syringe. A layer of bonding (G-Premio BOND, GC, Tokyo, Japan) was then applied to the enamel and dentine, left in place for 10 s, air-dried for 5 s to remove the excess solvent, and then light-cured for 10 s. Flowable BF composite or Ever x flow was then placed in the mesial and distal boxes and polymerized for 10 s from the occlusal surface. Then the matrix band was removed and the material was light-cured for 20 s on both buccal and lingual sides (3). A diamond bur (No. 8847KR, 314, 018, Komet, Lemgo, Germany) with the aid of a high-speed handpiece was utilized to finalize the shape of each cavity after DME, and the elevation level was checked with a millimeter-scale periodontal probe.

An intraoral scanner (Medit i700, Seoul, South Korea) was used to scan all the samples. The onlay restorations were designed using the Exocad software program (GmbH, Darmstadt, Germany). IPS E.max (CAD block HT; Ivoclar Vivadent, Schaan, Liechtenstein) was placed in the milling machine (ARUM Dentistry, Yuseong-gu Daejeon, South Korea) and milled in approximately 10–12 min. The restoration was separated from the block holder using a diamond-cutting instrument. Then, the restoration was crystallized and fired in one step, by placing it in the center of the IPS E.max CAD crystallization tray and crystallizing in a ceramic firing furnace (Programmed P310, Ivoclar Vivadent/technical, Schaan, Liechtenstein) for a 25 min firing cycle at 840 ºC according to the manufacturer’s instructions, which would be enough for the lithium disilicate crystals to grow in a controlled manner to obtain their final strength, shade, esthetic, and physical properties.

In respect to the cementation process, 9% hydrofluoric acid (Ivoclar Vivadent, Schaan, Liechtenstein) was applied to the intaglio surface of the onlay restoration for 20 s, then thoroughly rinsed with an air-water syringe and air dried. Subsequently, primer (G-Multi PRIMER, GC, Tokyo, Japan) was applied and dried with an air syringe (GC LinkForce manufacture instruction). Before cementation, the resin composite in groups with DME was pretreated with air-abrasion for 5 s using aluminum oxide powder with a particle size of 50 M (Dentify GmbH, Scheffelstr, Germany) (18) and 2.5 bars air pressure which was controlled with a manual pressure gauge, followed by extensive cleaning with water for 5 s using an air-water syringe (18). 37% phosphoric acid etch (Ivoclar Vivadent, Schaan, Liechtenstein) was then applied totally to the enamel, dentine, and gingival seal for 15 s, rinsed for 10 s and gently air-dried using a 3-in-1 syringe. The bonding (G-Premio Bond, GC, Tokyo, Japan) was then applied, left in place for 10 s and air-dried for 5 s to remove the excess solvent, and then polymerized for 10 s. Onlay restorations were cemented with G-CEM LinkForce (GC Europe, Tokyo, Japan) dual-cured resin cement which was extruded directly into the inner surface of the restoration and placed on the prepared tooth under a pressure of 1 kg for 2 min (21). The excess cement was then removed using 15c scalpel. The restoration margins were covered with an oxygen barrier solution (Ultra Dent, South Jordan, Utah) to prevent the formation of an oxygen-inhibited layer, then polymerized for 20 s on each of the occlusal, lingual, and buccal surfaces (3). Cemented restorations were stored in deionized water at 37 °C for 24 hours prior to thermocycling (22). The samples were then thermocycled for 500 cycles at 5 °C (± 2 °C) to 55 °C (± 2 °C), with a dwell time of 30 s. (23).

For the fracture resistance test, the samples were fixed in a metal holder and subjected to static loading using a universal testing machine (LARYEE Universal Testing Machine, Jinan, Shandong, China). A 6-mm-diameter custom-made stainless steel sphere was placed on the central fossa vertically to the long axis of the tooth to determine the fracture strength and the mode of failure (24). To prevent excessive load concentrations at specific locations on the tooth surface, a layer of aluminum foil with a thickness of 0.5 mm was positioned between the onlay surface and the steel sphere (24). The load was applied until fracture occurred at a cross-head speed of 0.5 mm/min, and the highest breaking load of each sample was recorded automatically in Newton (N) using a computer connected to the testing machine. A digital microscope at a magnification of 20x was used to assess the mode of failure following the specimen’s fracture (24). Each sample was examined from the five sides (occlusal, mesial, distal, buccal and lingual), and any visible fracture line was recorded. The modes of failure were identified and classified into three patterns (3): catastrophic failures where the fracture of the specimen would be in both restoration and tooth structure at or below acrylic resin or within root surfaces; combined fracture of both coronal tooth structure and restoration; and restoration fracture. Catastrophic failure types were considered non-favorable fractures, while combined fractures and restoration fractures were considered favorable fractures.

## Results

**Figure 3:**
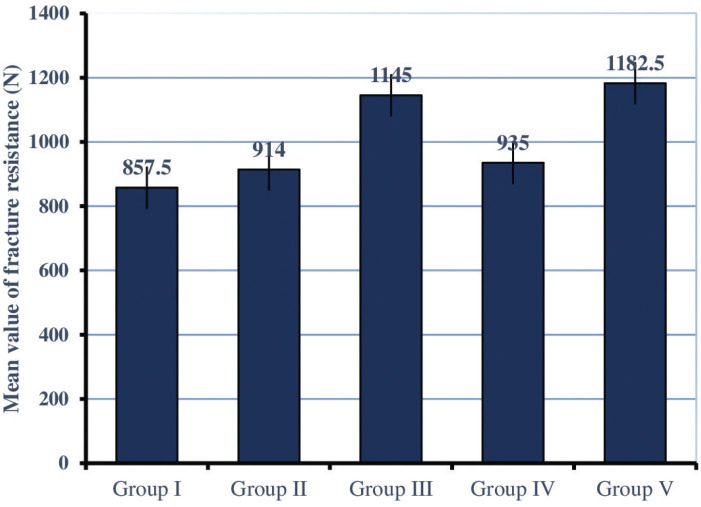
A bar chart graph shows the mean values (N) with the SE of fracture resistance for the study groups.

The values of fracture resistance are illustrated in Figure 3 which show, that teeth elevated 3 mm with SFRC (Group V) had the highest mean fracture resistance (1182.50 N) among the restored groups, while teeth without DME (Group I) had the lowest fracture resistance (857.50 N). In terms of DME levels, the ANOVA test showed a non-significant difference between teeth elevated 2 and 3 mm with flowable BF composite and those without DME (*p*=0.766). While there was a significant difference between 2 and 3 mm DME with SFRC and those without DME (*p*=0.014). The two independent sample t-tests revealed that the teeth elevated 2 or 3 mm with SFRC had higher resistance to fracture compared to those without DME, *p*=0.030 and 0.006, respectively. However, there was a non-significant difference between the two levels of marginal elevation with SFRC (*p*=0.737). In respect to the type of materials, teeth elevated with SFRC had significantly higher resistance to fracture compared to those elevated with flowable BF composite at both levels of 2 and 3 mm, *p*=0.041 and 0.038, respectively.

**Figure 4:**
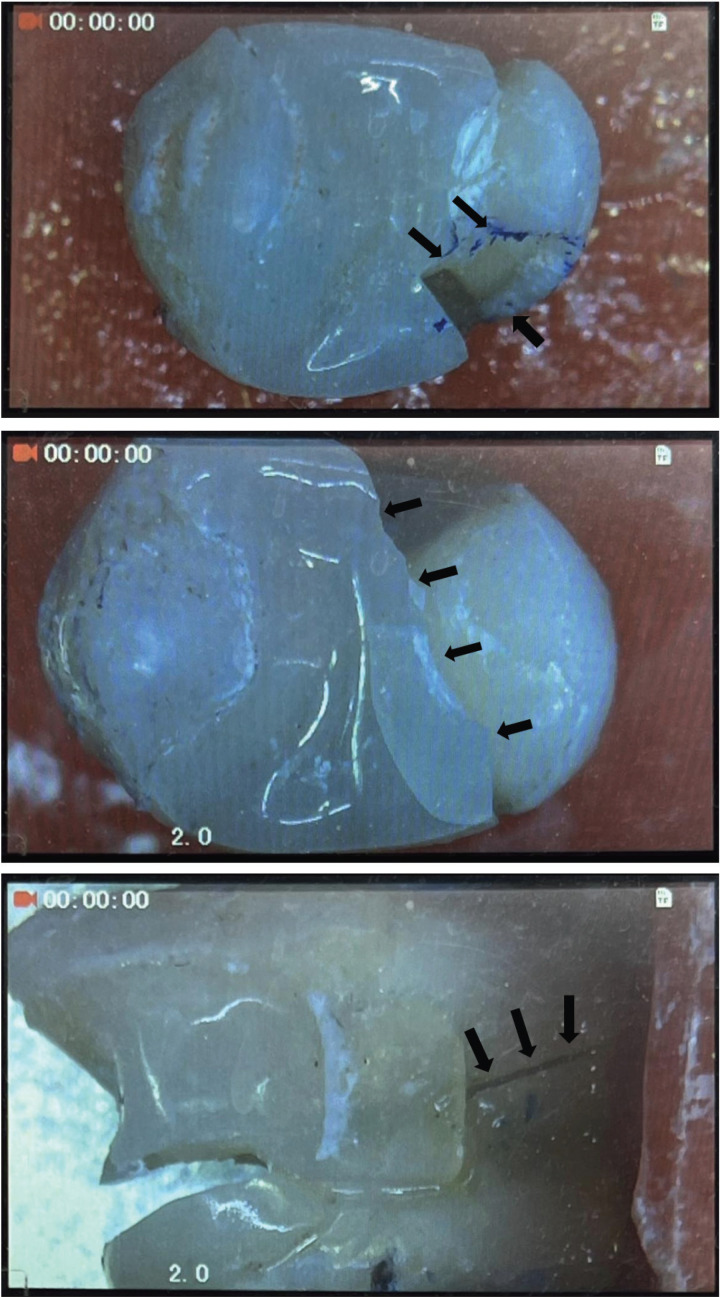
Type of fracture mode under a digital microscope (20x) is identified by the arrows: (a): Fracture of restoration; (b): Combined fracture of both restoration and tooth structure; (c): Catastrophic Failure.

Regarding the mode of fracture, the percentages of each type of fracture for all the study groups are illustrated in Table 2, and the modes of failure are shown in Figure 4. The highest unfavorable fracture was recorded for teeth without DME (70%, 7 out of 10 samples), whereas the lowest unfavorable fracture was recorded for the 3 mm DME with the SFRC group (30%, 3 out of 10 samples). Nevertheless, the Kruskal-Wallis test showed non-significant differences between the test groups regarding the level of marginal elevation and material types (*p*>0.05).

**Table 2:**
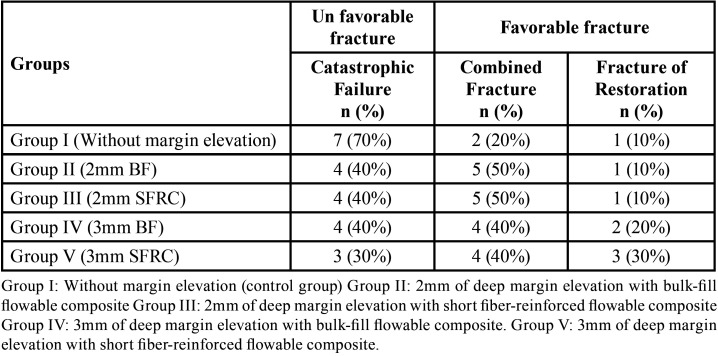
The distribution of modes of failure for the restored groups.

## Discussion

According to the findings of the present study, the first null hypothesis was rejected, where the fracture resistance of teeth elevated with SFRC and restored with a ceramic onlay had a higher fracture resistance compared to those elevated with flowable BF composite at both levels of marginal elevation, 2 and 3mm. However, there were a non-significant differences in the fracture resistance of ceramic onlay between 2 and 3mm DME of each material separately.

In this study, to simulate the caution action of the periodontal ligament during the application of the fracture resistance test, 0.2-0.3 mm of light body addition silicon was kept between the root and cold-cured acrylic resin (18). Maxillary first premolars were selected because studies have shown that they are more susceptible to fracture as their anatomical shape creates a tendency for separation of their cusps during mastication (25). In addition, clinically restored maxillary premolars might be subject to buccal and palatal strain as a result of occlusal loading (25). It was also reported that the incidence of maxillary premolar fractures is higher than that of mandibular premolars. Therefore, coronal restoration is necessary to support the remaining tooth structure (25).

Concerning the DME levels of the same restorative material type (BF and SFRC), the non-significant difference in fracture resistance of premolars elevated by 2 and 3 mm could be attributed to the fact that there was not a large difference (1 mm) in the bulk of the tooth structure that would affect the weakening/strengthening of the remaining tooth structure or ceramic restoration. Limited data were available comparing the effects of these two levels on the fracture resistance of premolars restored with a ceramic onlay. However, the findings revealed that the fracture resistance with 3 mm DME was slightly higher than that with 2 mm elevation with the same material type. This was expected due to the shorter cervical extensions of indirect restorations that were replaced with the restorative material which decrease the wedge action of the ceramic onlay on the tooth structure, i.e., have a positive effect on the tooth/restoration strength (19).

The non-significant difference between teeth elevated with flowable BF composite at both levels and those without DME might be due to the lower filler loading of Tetric n-flow BF composite, approximately 68.2 wt.% and 46.4 vol.%, that might not affect the strength of remaining tooth structure or ceramic restoration (26). A previous study showed that the filler loading of composites greatly influences their mechanical and physical properties (27). Similarly, another study found a positive relationship between filler loading and surface hardness (28). In addition, the type of Tetric n-flow BF composite filler which includes barium glass, ytterbium trifluoride, and copolymers wouldn’t affect the overall strength, a published study reported that composites containing barium glass filler showed significantly lower surface hardness (26). It can be speculated that all these factors might lead to a weak and unsTable restorative base (flowable BF composite) that negatively affects the strength of both the above ceramic onlay and the prepared tooth. The findings of the present study were in agreement with the findings of a previous study (3), which reported that the fracture resistance of ceramic onlays with BF composite used for DME did not significantly differ from those without marginal elevation; however, the authors of the above study used resin nano-ceramic for onlay restoration rather than lithium disilicate and BF conventional composite rather than BF flowable composite that had been used in the present study (3).

The fracture resistance of DME at both levels using SFRC was significantly higher than those without DME. This could be due to the higher fracture toughness of SFRC (15,16), which is provided by their composition and the presence of the microfibers that reinforce the tooth structure and act as stress transfer from the polymer matrix to the fibers, in addition to the behavior of individual fibers as a crack stopper (15). The findings of the present study were inconsistent with previous studies, which reported that DME did not significantly influence the fracture strength compared to teeth without DME. However, these studies applied different types of restorative materials for DME, including conventional composite, BF composite, glass ionomer cement, and resin-modified glass ionomer cement, which might affect the results (3,20,24). However, they agreed with another study who concluded that the fracture resistance of endocrown was improved by DME and the material utilized; and the fracture resistance with bulk-fill Smart Dentin Replacement DME was higher than those without marginal elevation (18).

Concerning the comparison between the two types of restorative materials used for DME in this study, the findings revealed that the material type influenced the fracture strength of restored premolars. The fracture resistance of teeth that received DME with SFRC was significantly higher compared to those elevated with flowable BF composite at both levels, 2 and 3 mm. This might also be due to the higher fracture toughness and flexural strength of SFRC compared to the flowable BF composite. Values for fracture toughness depend on the physical characteristics and chemical make-up of each component of restorative material, and material with a high fracture toughness could more effectively withstand crack initiation and propagation, consequently leading to an increase in fracture resistance (30). A previous study reported that SFRC exhibited significantly higher fracture toughness (2.8 MPa m1/2) and flexural strength (146.5 MPa) than flowable BF composites (*p*<0.05) (15), which is in agreement with the finding of the present study.

Regarding the mode of fracture, the results showed there was a non-significant difference in the mode of fracture among the restored groups; therefore, the second null hypothesis was accepted. These were in accordance with previous studies that found there was a non-significant difference in the mode of fracture between teeth with and without DME (3,20). However, the most irreparable fracture beyond the CEJ was recorded in teeth without DME (70%). This might be associated with increased occluso-gingival ceramic onlay thickness, where the deepest preparation could weaken the tooth structure drastically. These findings were in agreement with a previous study that compared the fracture resistance of feldspathic ceramic restorations with DME and those without marginal elevation and concluded that a longer occluso-gingival ceramic inlay height was more associated with catastrophic failure. However, the above study applied different types of material for DME, including glass ionomer, resin-modified glass ionomer, and enamel margin group, whereas in the present study, flowable BF and SFRC were applied for DME (12).

However, the 3 mm DME with SFRC recorded the most repairable fracture (70%). This, in addition to the shorter occluso-cervical height, could be attributed to the higher fracture toughness of SFRCs. The fracture toughness of a material is a measure of how well it prevents a crack or flaw from spreading under load. This property is provided by the random orientation of microfibers in the resin matrix and the formation of a fiber network, which seemed to have improved the material’s ability to resist fracture propagation and reduce the stress intensity at the crack tip, from which a crack propagates in an unstable manner and consequently induces a closure force on the crack by forming interlocking bridges. This would result in an increase in flexural properties and fracture toughness and lead to restorable rather than catastrophic failure (15,28)

According to the results of the present study, SFRC could be used as a restorative material for deep margin elevation. However, it is difficult to fully simulate the clinical condition, including dynamic cyclic fatigue; therefore, further studies are recommended *in vivo* setting to translate the results of this study.

## Conclusions

According to the results of this study, the following can be concluded.

1. The fracture resistance of premolars restored with ceramic onlays was not influenced by the levels of deep margin elevation whether it is 2 or 3mm.

2. Deep margin elevation with short fiber-reinforced flowable composites provided higher fracture resistance compared to both those without deep margin elevation and those elevated with bulk-fill flowable composites at both 2 and 3 mm marginal elevation.

3. Elevation of the deep cervical margin to 3 mm to reduce the thickness of ceramic restoration proximally, using short fiber-reinforced flowable composite, is recommended to reduce the chance of an unrestorable fracture.
